# Medical Chaos and Crime

**Published:** 1911-01

**Authors:** 


**Medical Chaos and Crime.** By **Norman Barnesby,** M.D. Octavo, pp. 384. London and New York: Mitchell Kennerly. 1910.

On the front cover it is stated that this book is, "An inquiry into the widespread demoralisation of the medical profession, a warning to the victimised public, and an earnest plea for immediate and drastic reform." The author says he offers testimony that will quickly convince the openminded reader, whether layman or physician, that a terrible problem confronts this nation, the neglect of which means such a wanton destruction of health and happiness and such an appalling loss of human life that one recoils from the spectacle. It is a book that will serve no useful purpose; it does not even state conditions fairly. Aside from a ma s of garbled reports and half sentences quoted, the writer offers no testimony that warrants any such sweeping denunciation of the profession of which he claims to be a member. In view of the fact that it is directed to the general public, it would indicate that the author expects a large sale from this source, which does sometimes follow a hysterical and sensational book of this character. He says he expects his statements will be challenged, but, like a good reformer, he will brave the storm as best he may. He takes himself too seriously. There will be no storm. If the writer succeeds in getting any one to give his book attention, it is much more than likely that his statements will be regarded as the vaporings of an incompetent, and a failure in his profession.

